# Ion permeation in potassium ion channels

**DOI:** 10.1107/S2059798320003599

**Published:** 2020-04-01

**Authors:** Leighton Coates

**Affiliations:** a Neutron Scattering Division, Oak Ridge National Laboratory, 1 Bethel Valley Road, Oak Ridge, TN 37831, USA

**Keywords:** ion channels, membrane proteins, X-ray crystallography, anomalous scattering

## Abstract

The study of ion channels dates back to the 1950s and the groundbreaking electrophysiology work of Hodgin and Huxley, who used giant squid axons to probe how action potentials in neurons were initiated and propagated. More recently, several experiments using different structural biology techniques and approaches have been conducted to try to understand how potassium ions permeate through the selectivity filter of potassium ion channels. Two mechanisms of permeation have been proposed, and each of the two mechanisms is supported by different experiments. The key structural biology experiments conducted so far to try to understand how ion permeation takes place in potassium ion channels are reviewed and discussed. Protein crystallo­graphy has made, and continues to make, key contributions in this field, often through the use of anomalous scattering. Other structural biology techniques used to study the contents of the selectivity filter include solid-state nuclear magnetic resonance and two-dimensional infrared spectroscopy, both of which make clever use of isotopic labeling techniques, while molecular-dynamics simulations of ion flow through the selectivity filter have been enabled by the growing number of potassium ion channel structures deposited in the Protein Data Bank.

## Abstract 2

Key structural biology experiments that have sought to elucidate how potassium ions permeate and pass through the selectivity filter of potassium ion channels are reviewed.

## 1. Introduction   

**Figure 1:**
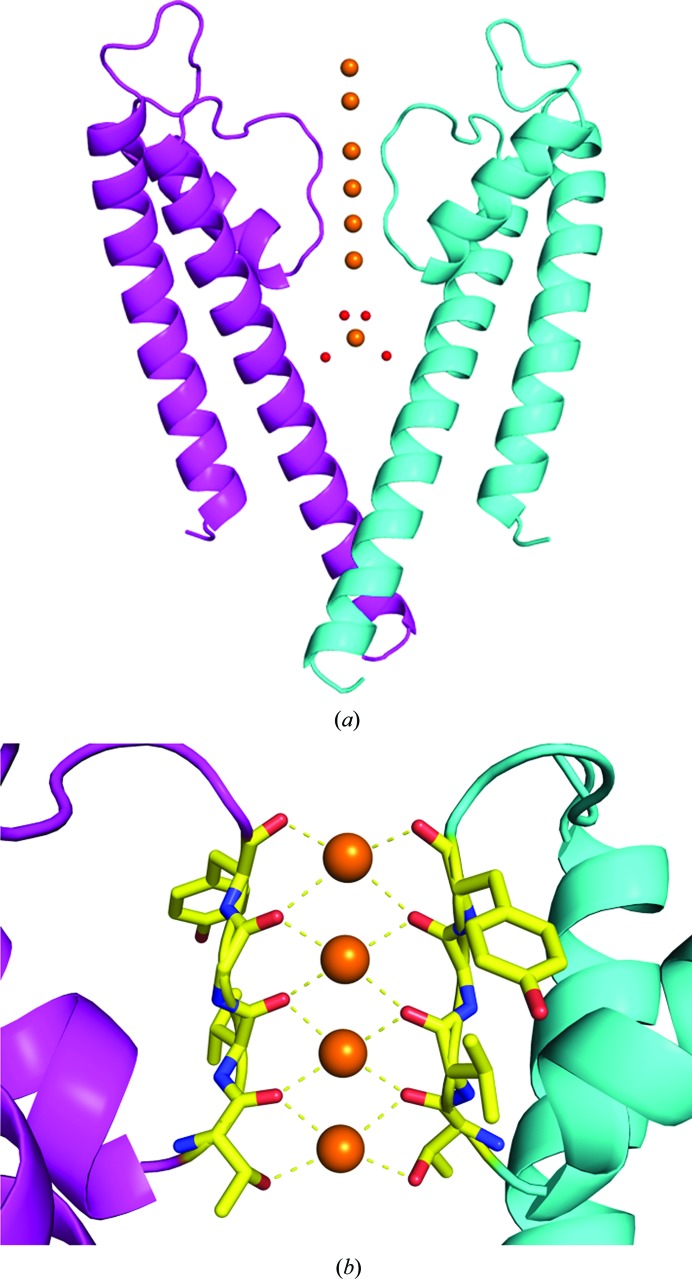
The structure of the potassium ion channel KcsA (PDB entry 1k4c). (*a*) The overall structure of KcsA is shown in cartoon format. For clarity, only two of the four subunits that make up a complete ion channel are shown in cyan and magenta, with potassium ions shown as orange spheres and water molecules shown as red spheres. (*b*) A close-up view of the selectivity filter (SF) of KcsA showing the four binding sites located within the SF. In the presence of four subunits, each binding site is coordinated to eight O atoms found in the backbone carbonyl atoms of Thr75, Val76, Gly77 and Tyr78 and the hydroxyl O atom of Thr75.

The most abundant cation found in the cytoplasm of living things is the potassium ion. Inside the cell the concentration of potassium ions is above 100 m*M*, while outside the cell the potassium ion concentration is usually less than 5 m*M* (Zhou *et al.*, 2001 ▸). Potassium ion channels are essential elements in cellular electrical excitability (Hille, 2001 ▸), while also maintaining a resting potential in non-excitable cells (Miller, 2001 ▸). Potassium ion channels selectively conduct potassium ions over sodium ions by a factor of 1000:1 (Hille, 2001 ▸). Genes that encode potassium channels are found in nearly all genomes, including those of bacteria and archaea. X-ray crystallography has revealed detailed crystal structures of prokaryotic potassium ion channels (Doyle *et al.*, 1998 ▸; Zhou *et al.*, 2001 ▸). The first structure of a potassium ion channel was that of KcsA (Doyle *et al.*, 1998 ▸; Zhou *et al.*, 2001 ▸; Fig. 1 ▸ *a*). This structure revealed several key structural motifs that are found within all potassium ion channels. Perhaps the most important of these is the selectivity filter (SF), which is a narrow channel running through the protein that is formed at the meeting point of four subunits that make up a complete ion channel (Fig. 1 ▸ *b*).

The amino-acid sequence of the SF (TVGYG) is conserved in potassium ion channels (Jiang *et al.*, 2002 ▸). The SF contains four discrete binding sites and was found to be around 4.5 Å wide: too narrow to permit the passage of hydrated potassium ions. However, the SF is lined by the carbonyl groups and a hydroxyl of the TVGYG residues from each of the four subunits that make up the complete ion channel (Fig. 1 ▸ *b*). Thus, each of the four binding sites within the SF is surrounded by eight O atoms that mimic the hydrated state of a potassium ion (Harding, 2002 ▸). This fundamental structure revealed how the selective permeation of potassium ions over sodium ions was achieved. However, the identity and population of the chemical species at each of the four binding sites within the SF could not be resolved from the 3.2 Å and later 2.0 Å resolution X-ray structures (Doyle *et al.*, 1998 ▸; Zhou *et al.*, 2001 ▸). The four binding sites within the SF are tightly packed, being only around 3.5 Å apart, raising the possibility of water molecules being found in the SF between potassium ions. As an X-ray crystal structure is an average of all of the molecules within the crystal, the relative occupancies of water and potassium ions at each of the four binding sites within the SF has been investigated using a range of techniques.

As potassium ion channels are integral membrane proteins, the resolution that can be obtained by X-ray crystallography is often limited to moderate resolutions; in the case of KcsA, the highest reported resolution structure so far is 1.90 Å (Zhou & MacKinnon, 2003 ▸). Semi-synthetic potassium ion channels such as NaK2K often diffract to a higher resolution. At the time of writing, the structure of NaK2K has been obtained to a resolution of 1.55 Å (Derebe *et al.*, 2011 ▸). Furthermore, ion channels are often formed from four sub­units with the SF found at their center. For this reason, they often crystallize in tetragonal-based space groups, often with the four binding sites in the SF on a unit-cell axis (Zhou & MacKinnon, 2003 ▸; Alam & Jiang, 2009*a* ▸). This means that all the atoms within the SF reside on special positions and have a maximum occupancy of 0.25. Like other tetrameric proteins in space group *I*4, merohedral twinning frequently occurs when crystallizing ion-channel proteins (Langan *et al.*, 2019 ▸; Alam & Jiang, 2009*a* ▸), and care must be taken to ensure that this type of twinning is detected and treated accordingly. Modern crystallographic data-reduction (Winn, 2003 ▸) and refinement programs (Adams *et al.*, 2010 ▸) have made the detection and treatment of twinned data much more routine (Padilla & Yeates, 2003 ▸; Yeates & Fam, 1999 ▸), but data from a nontwinned crystal are always more desirable. Luckily, with the advent of improved synchrotron beamlines and detectors, it is now possible to collect many data sets in a single shift to try and obtain data from untwinned crystals. Over the last 17 years, several key studies have probed how potassium ions permeate through the SF. In this article, we focus on structural biology techniques, but many biochemical (Hoomann *et al.*, 2013 ▸; Imai *et al.*, 2010 ▸) and molecular-dynamics studies (Flood *et al.*, 2019 ▸) have been conducted to measure ion and water flow through potassium ion channels. As might be expected, the results generated from multiple studies are not all in agreement with each other; several studies have supported a soft knock-on mechanism in which water molecules are co-transported along with potassium ions, while other studies have supported a hard knock-on mechanism in which only potassium ions are found in the SF. The key structural biology studies in this area are reviewed in order of publication from 2003 to 2019, with more studies expected in 2020 and the years beyond.

## 2. Anomalous X-ray scattering studies of KcsA   

X-ray crystallography-based anomalous scattering is a potent technique for determining macromolecular structures (Skubák *et al.*, 2004 ▸; Arndt *et al.*, 1982 ▸) and for identifying chemical species within a protein structure (Rocchio *et al.*, 2019 ▸). Anomalous scattering occurs when the wavelength or photon energy of the X-ray beam initiates changes in the quantum states of electrons within a particular type of atom (Skubák *et al.*, 2004 ▸). This anomalous scattering imparts unique properties to the structure factors collected that give unique information. With anomalous scattering, the amplitudes of pairs of structure factors called Friedel mates that are normally equal become different. This difference between Friedel mates is called an anomalous difference, and it arises only from the anomalously scattering atom, which in this case for KcsA is thallium. Thus, this technique can isolate the scattering from thallium and that from water even if they both occupy the same binding site within the SF.

Anomalous scattering was first used to investigate the contents of the SF of KcsA in 2003 (Zhou & MacKinnon, 2003 ▸). In this study, potassium was replaced with thallium. Thallium ions are among several ion species that are known to permeate potassium ions channels; they have a radius of 1.40 Å, which is very similar to the 1.33 Å radius of a potassium ion. Also, thallium has an anomalous scattering edge at 0.95 Å, which made it possible to reach the anomalous scattering edge on existing synchrotron beamlines at the time. A thallium ion possesses 80 electrons compared with the 18 found in a potassium ion and the ten electrons found in a water molecule. Thus, while the difference between a potassium ion and a water molecule is only eight electrons, the difference between a water molecule and a thallium ion is 70 electrons, making it easier to determine the relative occupancy of thallium ions within the SF, as the electron density for thallium ions will be much stronger and more concentrated than that for a potassium ion.

In this study, two approaches were used to determine the occupancy of thallium ions within the SF. In the first approach, structure factors (*F*_c_) were calculated based on the KcsA thallium structure and compared with the experimental data (*F*_o_). The *B* factors and occupancies of the thallium ions were then refined in separate rounds of refinement. A further correction was then applied to try to correct for a water molecule and a thallium ion being located at the same binding site within the SF according to 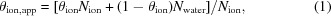
where *N*_ion_ and *N*_water_ are the number of electrons in a thallium ion (80) and the number of electrons in a water molecule (10), θ_ion,app_ is the apparent ion occupancy and θ_ion_ is the true ion occupancy. This approach estimated the thallium occupancy within the SF to be 0.75, with a 0.25 occupancy for a water molecule in the same position. Thus, of the four binding sites within the SF, three sites would be occupied with thallium ions and one site would be occupied with a water molecule.

In a second approach, single-wavelength anomalous difference (SAD) data were used to estimate the occupancies of thallium ions within the SF. Refinement of the SAD data estimated the thallium ion occupancy to be 0.63 at each of the four sites within the SF. This occupancy value was then used as a reference value to estimate the occupancy of potassium ions in the SF using a previous crystal structure of KcsA (Zhou *et al.*, 2001 ▸) crystallized with potassium ions. The potassium ion and thallium ion data sets of KcsA were placed on a common scale, and one-dimensional electron-density maps were sampled along the axis of the SF. These maps were calculated using *F*_o_ − *F*_c_ coefficients, where *F*_c_ was the inverse Fourier transform of a refined model in which the SF ions and protein atoms were omitted. Phases were obtained from a model refined in the absence of ions within the SF, with the SF harmonically restrained. In the one-dimensional electron-density map along the SF, the area under the peak present at each of the four binding sites within the SF was integrated for the potassium ion and thallium ion data sets. This integrated electron density was converted into an ion occupancy using (1) assuming that in the KcsA thallium structure the integrated electron-density value for each thallium peak within the SF represents the integrated electron density for a thallium ion with an occupancy of 0.63 and a water molecule with an occupancy of 0.37 according to (1). The estimated average occupancy for a potassium ion at each of the four sites within the SF was calculated using this process to be 0.53, with a water molecule occupancy of 0.47 at each of the four binding sites in the SF. Thus, the total number of potassium ions in the SF was calculated to be two, with two water molecules within the SF.

**Figure 2:**
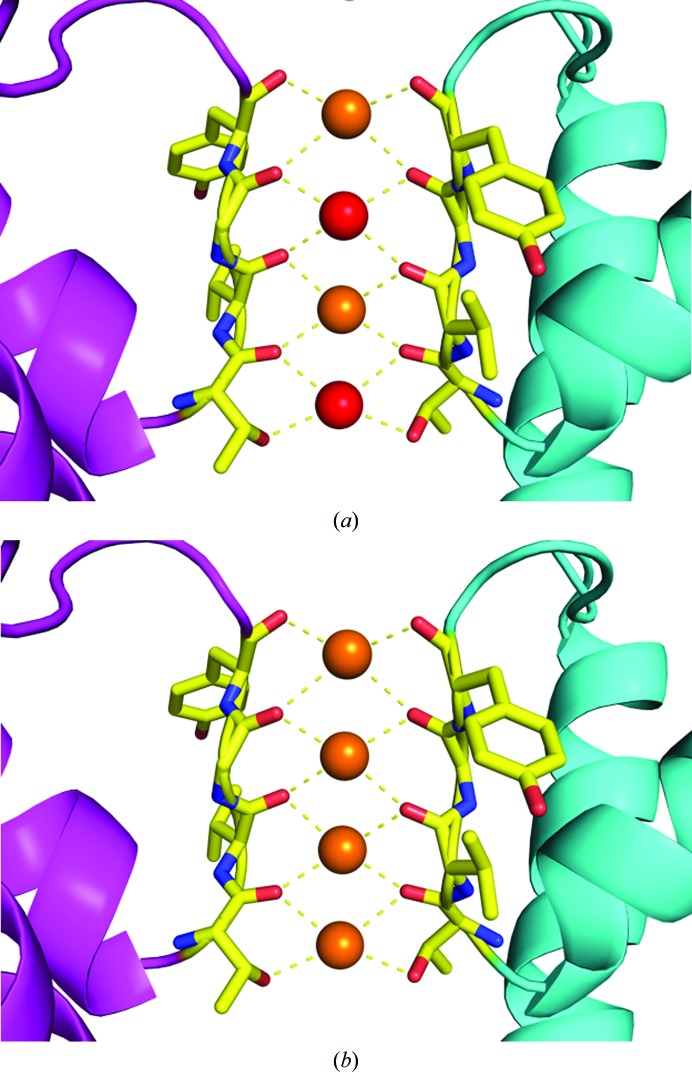
The two proposed mechanisms by which potassium ions permeate through potassium ion channels. (*a*) In the soft knock-on mechanism, potassium ions (orange spheres) are separated by water molecules (red spheres) in the SF. (*b*) In the hard knock-on mechanism, potassium ions (orange spheres) occupy each of the four binding sites within the SF.

These experiments agreed with earlier electrophysiology measurements of the ‘streaming potential’, in which the data suggested that one water molecule accompanied each potassium ion through the ion channel (Alcayaga *et al.*, 1989 ▸). Later on, this mechanism became known as the ‘soft knock-on’ mechanism in which potassium ions are separated by water molecules as they pass through the SF (Fig. 2 ▸ *a*).

## 3. Reinterpretation of previous anomalous X-ray data and molecular-dynamics studies   

This water-mediated soft knock-on or streaming potential mechanism was the generally accepted model in the field until 2014, when a new study suggesting that the ions are in direct contact with each other and that no water molecules are involved in the conduction process was published (Köpfer *et al.*, 2014 ▸). This study was in part enabled by the Protein Data Bank (PDB; Burley *et al.*, 2019 ▸), a valuable resource for the worldwide scientific community with its catalog of deposited protein structures and corresponding structure factors. This enables researchers years later to use new ideas, techniques and software to re-examine previous data and structures that often required large amounts of publicly funded resources to obtain. Anomalous scattering data from KcsA and other potassium ion channels were re-examined using *SHELXD* (Sheldrick, 2010 ▸) and *SHELX* (Sheldrick, 2015 ▸), and the occupancies of the anomalously scattering atoms within the SF were refined. The results of the *SHELX* refinements on several sets of anomalous diffraction data showed that each of the four sites within the SF was fully occupied with metal ions. Molecular-dynamics simulations of ion flow through the SF of KcsA within the same study (Köpfer *et al.*, 2014 ▸) also suggested that water molecules were absent from the SF (Fig. 2 ▸ *b*) and that direct ionic contacts between adjacent potassium ions within the SF were not energetically prohibitive. Instead, they serve to increase ion flux to the maximum achievable speed through the SF.

## 4. Two-dimensional infrared spectroscopy   

In 2016, the inherent high time resolution of 2D infrared spectroscopy coupled with a specially labeled KcsA protein was used to probe the contents of the SF (Kratochvil *et al.*, 2016 ▸). In this, a specially constructed KscA protein with ^13^C,^18^O labeling on the backbone carbonyls of Val76, Gly77 and Gly79 within the SF was produced to isolate and probe the contents of the SF spectroscopically. As atomic bond vibrations are sensitive to their electrostatic environment, their frequencies are influenced by nearby ions and water. 2D infrared spectra were recorded from the ^13^C,^18^O-labeled KcsA sample and an unlabeled KcsA sample. A difference spectrum was then generated by subtracting the spectrum of the un­labeled KcsA sample from that of the labeled KcsA spectrum. Molecular-dynamics simulations then computed 2D infrared spectra for all relevant ion configurations. This study concluded that water must be present in the SF to reproduce the experimental 2D line shapes that were measured. Further 2D infrared spectroscopy studies in which different backbone carbonyls were labeled would have been of great interest. However, subsequent 2D infrared spectroscopy and molecular-dynamics studies in 2018 showed that the 2D infrared spectra produced from KcsA are also in accordance with the direct knock-on mechanism (Kopec *et al.*, 2018 ▸).

## 5. Anomalous X-ray scattering from potassium ions   

**Figure 3:**
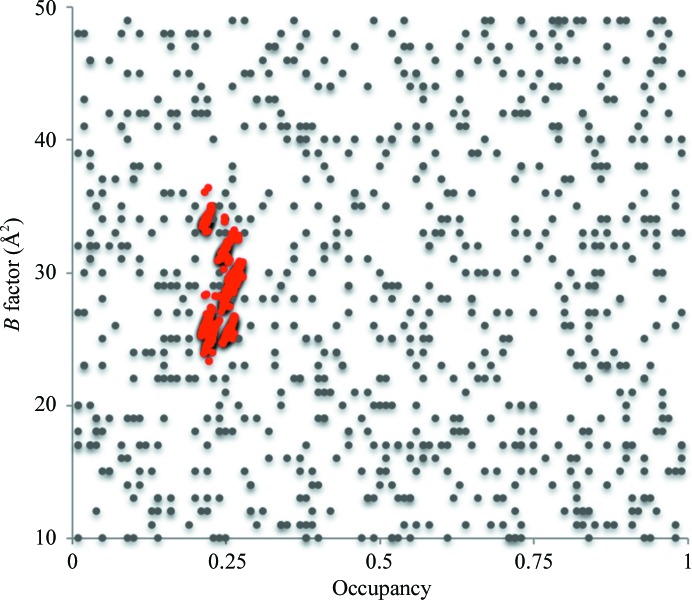
Results of occupancy refinement for the potassium ions within the SF using anomalous data. 100 starting models were generated with initial occupancies and ADP values for potassium ions randomly drawn from the ranges 0–1 and 10–50 Å^2^, respectively, for each of eight potassium ions per structure (gray dots). The refined potassium occupancy values for each potassium ion (red dots) cluster around 0.25, which represents the maximum occupancy for these atoms as they lie on special positions within space group *I*4.

In 2017, a long-wavelength macromolecular crystallography beamline (I23) started operation at Diamond Light Source, UK (Wagner *et al.*, 2016 ▸). This beamline is optimized to collect data at wavelengths of up to 5.9 Å, meaning that the anomalous scattering edge of potassium (3.45 Å) could be reached for data collection for the first time. The sodium–potassium (NaK) ion channel from *Bacillus cereus* forms a nonselective SF which allows the transport of potassium and sodium ions through the channel (Shi *et al.*, 2006 ▸). The NaK channel shares a high amino-acid sequence homology and a similar structure with the bacterial potassium ion channel KcsA (Alam & Jiang, 2009*b* ▸), but their SFs adopt different conformations. Mutation of two residues, Asp66Tyr and Asn68Asp, in the NaK amino-acid sequence causes main-chain conformational changes in the SF (Derebe *et al.*, 2011 ▸). This mutant possesses a TVGYG SF sequence and the SF is almost identical (r.m.s.d. of 0.16 Å) to that found in the potassium ion channel KcsA and is highly selective for potassium ions (Derebe *et al.*, 2011 ▸). The removal of the first 19 amino acids from the wild-type NaK sequence, which form the interfacial helix, places the ion channel in a wide-pore conformation (Alam & Jiang, 2009*a* ▸). This mutant is named NaK2K and is often used as a model system for understanding selective potassium channels as its SF is identical to that found in KcsA (Alam & Jiang, 2009*a* ▸; Langan *et al.*, 2019 ▸). We recently conducted anomalous scattering studies (Langan *et al.*, 2018 ▸) of the potassium ions within the SF of NaK2K at a wavelength of 3.35 Å. Using the anomalous difference data, we were able to refine the occupancy of the potassium ions within each of the four binding sites within the SF using the *phenix.refine* software package (Adams *et al.*, 2010 ▸). To test the stability and reproducibility of the potassium ion occupancy and *B*-factor refinements, we generated 100 starting models using *phenix.pdb_tools* from the *Phenix* suite. These 100 starting models had initial occupancies and *B*-factor values for the potassium ions within the SF randomly drawn from the ranges 0–1 and 0–50 Å^2^, respectively, for each potassium ion per structure. The crystallo­graphically refined potassium ion occupancy values clustered around 0.25 (Fig. 3 ▸). This corresponds to each of the four binding sites within the SF being fully occupied with potassium ions, giving a total of four potassium ions within the SF. This work is in agreement with the studies of Köpfer *et al.* (2014 ▸), but is in disagreement with the earlier anomalous diffraction studies conducted on KcsA (Zhou & MacKinnon, 2003 ▸) in which the estimated total number of potassium ions within the selectivity filter was two.

## 6. Solid-state nuclear magnetic resonance studies of the SF contents   

In 2019, solid-state nuclear magnetic resonance (NMR) experiments (Oster *et al.*, 2019 ▸) using fully deuterated, ^13^C,^15^N-labeled NaK2K protein investigated the contents of the SF. This experiment differs slightly from the crystallo­graphic experiments in that the specially labeled NaK2K protein was inserted into a membrane rather than being solubilized in detergent, meaning that the protein is in an environment closer to its native state. This specially labeled deuterated protein was exposed to H_2_O. H and D atoms that are attached to polar atoms such as O and N atoms readily exchange in solution by a process called H/D exchange that is often used in neutron diffraction studies (Vandavasi *et al.*, 2016 ▸; Tomanicek *et al.*, 2013 ▸). The deuterated NaK2K protein was placed in an H_2_O solution, meaning that any solvent-exposed D atoms on polar atoms would quickly exchange for H atoms, making these exchanged sites visible to NMR. If water molecules (H_2_O) can enter the SF, then H/D exchange of exchangeable D atoms on amino-acid residues that make up the SF would be expected to take place, which was not observed. The role of the conserved water molecules behind the SF and H/D exchange was a complicating factor. However, a similar result was also obtained using water-to-protein magnetization-transfer experiments. These experiments showed that no water molecules could be located in the central binding sites of the SF as long as potassium ions were present at physiological concentrations.

## 7. Possible future studies   

**Figure 4:**
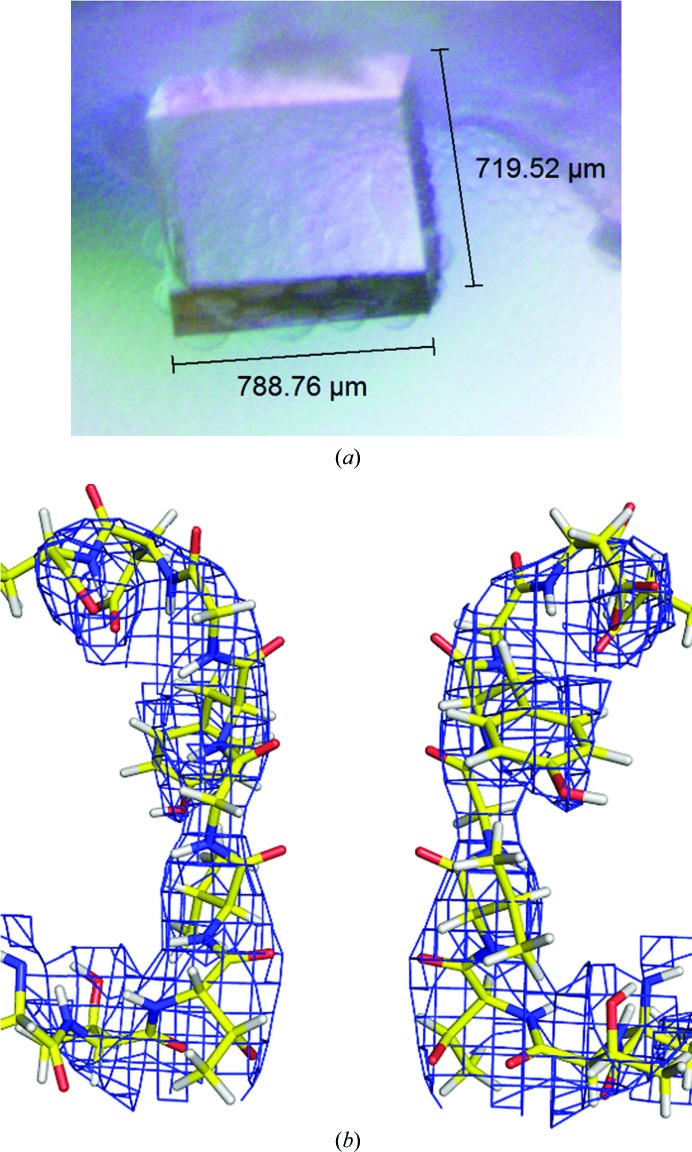
Neutron diffraction studies of NaK2K. (*a*) A large crystal of NaK2K formed from hydrogenated protein produced neutron diffraction data to 3.50 Å resolution. (*b*) A low-resolution (3.5 Å) 2*F*_o_ − *F*_c_ neutron density map at 1σ of the NaK2K selectivity filter is shown as a blue mesh; higher resolution data will be needed to probe the contents of the SF.

In neutron diffraction, heavy-water (D_2_O) molecules and potassium ions have very different scattering profiles. In a heavy-water molecule all three atoms possess a similar scattering power, giving a boomerang-shaped neutron density peak (Afonine *et al.*, 2010 ▸), while potassium scatters neutrons with a scattering power of about half of those of the O and D atoms found in a heavy-water molecule. Thus, neutron protein crystallography is more sensitive to water molecules than potassium ions, which is the reverse of what is found in X-ray crystallography. Neutron diffraction data have been collected from from a large crystal of hydrogenated NaK2K (Fig. 4 ▸ *a*) using the MaNDi instrument at the Spallation Neutron Source (SNS; Coates *et al.*, 2015 ▸) to a resolution of 3.50 Å (Langan *et al.*, 2019 ▸). Unfortunately, on producing initial neutron maps of NaK2K (Fig. 4 ▸ *a*) the resolution of the diffraction data was not high enough to allow visualization of the atoms within the SF. However, work is under way to produce crystals using perdeuterated NaK2K. This will help to reduce the background emanating from the sample by a factor of 40 (Coates *et al.*, 2014 ▸), enabling higher resolution data to be collected.

## 8. Conclusions   

Many ingenious experiments using different techniques and approaches have been used to try to understand how potassium ions permeate through the SF. Several studies have shown that the transport of potassium ions uses a hard knock-on mechanism, while other studies have shown that transport occurs via a soft knock-on mechanism. It is unlikely that a consensus will be reached any time soon. However, it is reassuring to know that the data from the protein crystallo­graphy studies described here are freely available in the PDB (Burley *et al.*, 2019 ▸) to this and future generations of scientists.
